# Effects of Brazilian Pepper Tree (*Schinus terebinthifolius* Raddi) Ethanolic Leaf Extract on Growth Performance and Expression of Intestinal Immune-Related Genes in Nile Tilapia (*Oreochromis niloticus*)

**DOI:** 10.3390/biology15060476

**Published:** 2026-03-15

**Authors:** Eman Mohamed, Mahmoud Mostafa Mahmoud, Yosra M. I. El Sherry, Amr Abdullah, Soad A. L. Bayoumi, Rofida Wahman, Abeer M. Mahmoud, Mahmoud M. S. Farrag, Ebtsam Sayed Hassan Abdallah

**Affiliations:** 1 Department of Aquatic Animal Medicine and Management, Faculty of Veterinary Medicine, Assiut University, Assiut 71529, Egypt; emanmohamed@aun.edu.eg (E.M.); ebtsamsayed@aun.edu.eg (E.S.H.A.); 2 Department of Fish Diseases, Faculty of Veterinary Medicine, Aswan University, Sahari, Airport Way, Aswan 81528, Egypt; yosramohamed@vet.aswu.edu.eg; 3 Department of Architectural Engineering, Imam Mohammad Ibn Saud Islamic University (IMSIU), Riyadh 11432, Saudi Arabia; asabdullah@imamu.edu.sa; 4 Pharmacognosy Department, Faculty of Pharmacy, Assiut University, Assiut 71526, Egypt; soad.bayoumi@pharm.aun.edu.eg (S.A.L.B.); rofidawahman@aun.edu.eg (R.W.); 5 Pharmacognosy Department, Faculty of Pharmacy, Badr University in Assiut (BUA), Assiut 71526, Egypt; 6 Division of Endocrinology, Department of Medicine, University of Illinois Chicago, Chicago, IL 60612, USA; 7 Department of Kinesiology and Nutrition, College of Applied Health Sciences, University of Illinois Chicago, Chicago, IL 60612, USA; 8 Zoology Department, Faculty of Science, Al-Azhar University, Assiut 71524, Egypt; mahmoudfarrag42@azhar.edu.eg

**Keywords:** *Schinus terebinthifolius* Raddi, leaf ethanolic extract, growth performance, intestinal immune-related genes, cichlids

## Abstract

This study investigated the ethanolic leaf extract of Brazilian pepper tree (*Schinus terebinthifolius* Raddi) for its metabolite composition and effects on growth performance and intestinal immune gene expression in Nile tilapia (*Oreochromis niloticus*). Ultra-performance liquid chromatography-electrospray ionization-mass spectrometry (UPLC-ESI-MS/MS) in positive and negative modes revealed a diverse profile of 33 peaks in each polarity, identifying key compounds such as phenolic acids (e.g., gallic acid and ferulic acid), flavonoids (e.g., myricetin-O-glucoside and quercetin 3-O-glucoside), gallotannins (e.g., glucogallin and pentagalloylglucose), and triterpenoids (e.g., masticadienoic acid). A 60-day feeding trial with four groups (control and three extract doses; 0.5%; T0.5%, 1%; T1% and 2%; T2%) demonstrated dose-dependent enhancements in growth metrics, where final body weight increased by up to 106.9 ± 3.6 g, weight gain% by 197.3 ± 3.5%, and the growth rate got more than doubled in T2% (2.4 ± 0.1), alongside improved feed conversion ratio (1.24 ± 0.01) at 30 days and condition factor (stabilized at 2.1 ± 0.0) at 60 days post-feeding. Viscero- and gastro-somatic indices declined insignificantly in most feed groups, indicating improved muscle growth. Biphasic patterns were observed in intestinal gene expression as follows: over 60 days, the *IL-1β* gene upregulated at low doses but returned to normal at high doses. The *IL-10* gene upregulated progressively, promoting an anti-inflammatory balance. In fish fed medium and high doses (T1% and T2%), the *IgM* gene is upregulated, supporting humoral immunity. These outcomes, linked to the extract’s previously described antioxidants, anti-inflammatory, and antimicrobial bioactive compounds, suggest that *S. terebinthifolius* is a promising natural feed additive for sustainable tilapia aquaculture and warrants further validation for commercial application.

## Simple Summary

This study examined a natural leaf extract from the Brazilian pepper tree and its effects on Nile tilapia growth and gut health. Chemical analysis showed the extract contains beneficial compounds such as phenolics, flavonoids, tannins, and triterpenoids. In a 60-day feeding trial, fish given higher doses of the extract grew faster, gained more weight, and used feed more efficiently than controls, with reduced body fat. The extract also influenced immune-related genes, increasing anti-inflammatory signals and immune protection at appropriate doses. Overall, the results suggest this plant extract could be a safe, natural feed additive to improve growth, immunity, and sustainability in tilapia aquaculture.

## 1. Introduction

Chemotherapy has traditionally been used in aquaculture to prevent and control diseases. However, chemical treatments can have significant adverse effects on both the environment and human health. These effects include the development of resistant bacterial strains and the accumulation of residues in tissue. Recently, there has been a shift towards using plant products to control aquaculture diseases, rather than chemical medicines [1].

Natural products, especially medicinal plants and their derivatives, are increasingly recognized as viable and sustainable substitutes for antibiotics in aquaculture. This transition addresses the challenge of bacterial resistance while satisfying consumer preferences for residue-free food. Incorporating these phyto-therapeutic agents for disease management enhances the sustainability of tilapia production, minimizes environmental footprints, and conforms to market demands for safer, higher-quality food products [2]. The chemical properties of essential oils directly influence animal health by modulating metabolism, intestinal microbiota, and immune function [2]. In aquaculture, the volatile constituents of plant-derived essential oils enhance fish growth and immunity [3]. Studies have shown that plant products can enhance feed intake, promote weight gain, strengthen immune responses, and have antibacterial and antiparasitic properties in fish and shellfish aquaculture. This is due to active molecules like alkaloids, terpenoids, saponins, and flavonoids [1].

*Schinus terebinthifolius* Raddi, commonly known as Aroeira in Brazil, belongs to the *Anacardiaceae* family and is widely found throughout Brazil, primarily in the Atlantic Forest. *S. terebinthifolius* is also present in tropical and semitropical regions of Africa and the United States [4]. In Brazil, the leaves are applied topically to treat wounds and promote tissue healing. This species possesses a broad spectrum of pharmacological properties, including antimicrobial, anti-inflammatory, antioxidant, anticancer, antiulcerogenic, and tissue-repairing effects [5,6,7,8]. Additionally, they are often used as an infusion to treat rheumatism, oral candidiasis, and infections of the respiratory, digestive, and urinary systems [9,10,11]. Topical application of the essential oil of *S. terebinthifolius* leaf is effective against respiratory problems and fungal and candidal infections. Its effectiveness is attributed to its high monoterpenes concentrations [12]. It is effective against *Aspergillus niger*, *A. parasiticus*, and *Candida albicans*. Additionally, it exhibits antibacterial activity against *Streptococcus agalactiae*, *Shigella dysenteriae*, *Pseudomonas aeruginosa*, *Escherichia coli*, *Bacillus subtilis*, *Staphylococcus albus*, *S. aureus*, and *S. intermedius* [13,14,15]. The antifungal and antibacterial effects of the ethanolic and dichloromethanic leaf extracts against *E. coli*, *P. aeruginosa*, *S. aureus* and *C. albicans* may be due to the presence of secondary metabolites such as phenols, flavones, flavonoids, xanthones, leucoanthocyanidins, flavanones and free steroids [14,15,16]. Among 23 extracts derived from 12 Cuban plants, the aqueous extract from *S. terebinthifolius* leaves demonstrated the strongest inhibitory effect against *S. aureus* and also suppressed the growth of *B. subtilis* [9]. Extracts from the leaves and essential oils derived from the fruits of *S. terebinthifolius* effectively kill *Stegomyia aegypti* and *Aedes aegypti* larvae [17,18]. Alpha-pinene, extracted from the leaves, has shown high potential to induce apoptosis in melanoma cancer cells [19]. Gallic acid and methyl gallate, the major components of the acetate fraction, have exhibited important anti-allergic properties [20]. Lectin extracted from the leaves has shown strong antimicrobial activity with bactericidal and fungicidal properties [21].

The Nile tilapia (*Oreochromis niloticus*), a freshwater fish native to Africa, is now farmed globally. It is the world’s third most-produced aquaculture species, and its farming has seen immense growth over the past twenty years. It plays a crucial role in improving food security in many developing countries [22,23]. As the most commonly farmed tropical fish group worldwide, total tilapia production reached approximately 4.5 million tons in 2013 and is projected to surpass 6.6 million tons by 2030 [24]. Egypt is now the third worldwide tilapia producer, following China and Indonesia, and the first among African countries, by producing about 71% of African tilapia production [25]. The country’s economy and food security greatly benefit from tilapia aquaculture [26]. The vital role played by aquaculture to fulfil the growing demand for fish as a source of protein has driven its rapid growth, expansion, and intensification of the industry. However, this acceleration also raises the risk of disease outbreaks as fish experience increased stressors. Since there is limited literature investigating the effect of *S. terebinthifolius* ethanolic leaf extract on *O. niloticus*, the present study was conducted to examine the impact of various concentrations of *S. terebinthifolius* ethanolic leaf extract on *O. niloticus* growth parameters and the expression of intestinal immune-related genes.

## 2. Materials and Methods

### 2.1. Ethics Statement

The methods used in the in vivo experiments were approved (Code No. 06/2025/0319), and the fish used in this study were handled and treated strictly in accordance with procedures outlined in the Guide for the Use of Experimental Animal Welfare Committee of the Faculty of Veterinary Medicine, Assiut University, Assiut, Egypt. The fish were not subjected to unnecessary pain or sacrifice.

### 2.2. Plant Oil Extraction

Leaves of *S. terebinthifolius* Raddi were collected during the flowering stage in 2024, from the Assiut University Campus, Assiut, Egypt. The plant was authenticated by Dr Mostafa Abo el ela, Botany Department, Faculty of Science, Assiut University. Voucher specimen was kept in the herbarium, Pharmacognosy Department, Faculty of Pharmacy, Assiut University. The air-dried powdered leaves (1 Kg) were exhaustively extracted with 70% ethanol by maceration at room temperature (4 × 2 L), and the extract was filtered using Whatman No. 1 filter paper. The ethanolic extract was concentrated under reduced pressure using a rotary evaporator at 40 °C to obtain a viscous residue, which is alcohol- free (187.2 g). The extract was preserved at minus twenty Celsius until it could be used. Immediately before its application, this residue was redissolved in 70% ethanol in order to achieve the required working concentrations (0.5%, 1% or 2%), which was then sprayed onto the fish feed.

### 2.3. Determination of the Chemical Profile of the Extract

The ethanolic extract derived from *S. terebinthifolius* leaves was subjected to qualitative profiling utilizing ultra-performance liquid chromatography interfaced with electrospray ionization mass spectrometry (UPLC-ESI-MS/MS; Waters Corporation, Milford, MA, USA). Analyses were conducted on a Waters ACQUITY UPLC system equipped with a quaternary solvent manager (QSM), flow-through needle autosampler (FTN), and XEVO TQD triple quadrupole instrument (Waters Corporation, Milford, MA, USA) mass spectrometer. ESI-MS positive and negative ion acquisition modes were employed for comprehensive metabolite detection. The chromatographic separation was achieved on an ACQUITY UPLC BEH C18 column (Waters Corporation, Ireland; 2.1 mm × 100 mm, 1.7 μm particle size) operated at ambient temperature.

The mobile phase consisted of solvent A (water containing 0.1% formic acid) and solvent B (acetonitrile containing 0.1% formic acid). A linear gradient elution was employed at a constant flow rate of 0.2 mL/min as follows: 0–2 min, 90% A; 2–5 min, 90–70% A; 5–15 min, 70–30% A; 15–22 min, 30–10% A; 22–25 min, 10% A; 25–26 min, 10–0% A; 26–29 min, 0% A; and 29–32 min, 0–90% A. The injection volume was 1.0 μL.

Mass spectrometric detection was performed in both positive and negative ionization modes (ESI+ and ESI−) over a scan range of m/z 100–1000. For ESI+ mode, the instrument parameters included a capillary voltage of 3.04 kV, cone voltage of 30 V, extractor voltage of 3 V, source temperature of 150 °C, a desolvation temperature of 400 °C, cone gas flow of 50 L/h, and desolvation gas flow of 600 L/h. For ESI- mode, the parameters were as follows: capillary voltage, 3.09 kV; cone voltage, 30 V; extractor voltage, 3 V; source temperature, 150 °C; desolvation temperature, 400 °C; cone gas flow, 50 L/h; and desolvation gas flow, 600 L/h. Data acquisition and processing were managed using MassLynx software (version 4.2), with OpenLynx (version 4.2) for automated peak detection and integration.

### 2.4. Experimental Fish and Rearing Facilities

Apparently healthy *O. niloticus* (n = 240) were bought from a private fish farm in the Assiut governorate. The fish were transported to the Wet Lab of the Department of Aquatic Animal Medicine at Assiut University’s Faculty of Veterinary Medicine. Fish were maintained in 500 L flow-through tanks and allowed to acclimate to laboratory conditions for two weeks, according to the protocol for maintaining bioassays by Ellsaesser and Clem [27]. Fish were fed on floating pellets (Skretting, Egypt, Table 1). The analytical specifications of the diet are: crude protein 30%, fat 6%, crude fibre ≤ 5.29, and metabolic energy is 3990 Kcal/kg fish feed, as described by the producer.

### 2.5. Experimental Design

**Figure 1:**
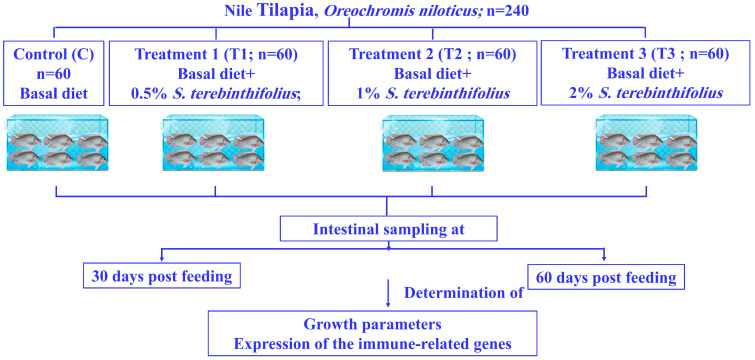
Diagram illustrating the experimental design.

Acclimated *O. niloticus* (average weight of 35.1 ± 0.9 g and total length of 12.3 ± 0.6 cm) was randomly divided into the following four groups: control group (C), treatment group 1 (T0.5%), treatment group 2 (T1%), and treatment group 3 (T2%). Each group was composed of three separate replicates, with 20 fish in each replicate in 500-litre tanks. The control group (C) was fed on a basal diet, while treatment groups T0.5%, T1%, and T2% were provided with the same diet supplemented with 0.5%, 1%, and 2% of the *S. terebinthifolius* leaf extract, respectively (Figure 1). The ethanolic leaf extract was applied as a surface coating by spraying 200 mL of the respective concentration solution (0.5, 1, or 2% prepared in 70% ethanol) onto each kilogram of basal diet pellets, followed by thorough mixing and air-drying at room temperature until constant weight was achieved. The control basal feed was sprayed with the same amount (200 mL/kg fish feed) of 70% ethanol. No inert carrier (e.g., α-cellulose or microcrystalline cellulose) was used to compensate for extract addition. This method ensured that the basal nutrient composition remained identical across all diets, with the extract adhering to the pellet surface rather than replacing any feed component. The extract inclusion levels were kept low (0%, 0.5%, 1.0%, and 2.0% *w*/*w*), and the non-nutritive nature of the ethanolic solvent (which was fully evaporated), any potential dilution effect, was considered negligible compared to the bioactive influence of the extract. This approach is consistent with published studies in fish feed trials [28]. It was preserved at minus twenty until used. Fish were fed twice daily (3% of their body weight for up to 60 days). Survival rates were recorded throughout the feeding period (60 days).

At biweekly intervals, every fish tank was measured for its biomass and population count. The daily feeding amounts were adjusted in response to changes in the overall biomass. During the entire experiment, the mean values of water temperature, oxygen concentration, and acidity (pH) were consistently monitored. The average water temperature, dissolved oxygen levels, and pH levels were 22.0 ± 0.6 °C, 6.0–7.0 mg/L, and 7.2–7.5, respectively. The photo period was maintained on a 12:12 h light/dark schedule. The feeding trial lasted for 60 successive days, and samples were collected at 30 d and 60 d post-feeding. Nine fish from each group (C, T0.5%, T1%, and T2%) were randomly caught at each checkpoint (30 d and 60 d). The fish (n = 9/group/checkpoint) were euthanized using eugenol [29]. In each checkpoint (30 d and 60 d), samples from the anterior intestines of three fish per tank were pooled, preserved in *RNA* later (Applied Biotechnology, Ismailia, Egypt), and kept at −80 °C until RNA extraction. Feed was discontinued one day before intestinal sampling.

### 2.6. Feed Efficiency, Growth Performance and Somatic Indices

At the beginning of the experiment, the initial body weight (IBW) and total length (TL) of each fish were recorded. After 30 days of feeding, measurements including final body weight (FBW), total length, and gut weight (GW) were recorded for every fish in each group to evaluate the dietary effects. Additionally, calculations for feed efficiency, growth performance, and somatic indices were performed according to Aanyu [30] and Bodin, et al. [31] using the specified formulas provided as follows:

Weight gain (WG) = final body weight (g) − initial body weight (g).

Weight gain percentage (%WG) = 100 × ((final body weight (g) − initial body weight (g))/initial body weight (g)).

Feed intake (FI) = total feed consumed by fish during the feeding period (g)/total number of fish.

Feed conversion ratio (FCR) = feed intake (g)/weight gain (g).

Growth rate (GR) = weight gain (g)/duration of feeding period in days (d).

Specific growth rate (SGR) = 100 × ((ln (final body weight) − ln (initial body weight))/duration of feeding period in days (d)), where Ln = the natural log.

Condition factor (CF) = 100 × (final body weight (g)/final total length (cm)^3^).

Viscero-somatic index (VSI) = 100 × (visceral weight/final body weight (g)).

Gastro-somatic index (Ga-SI) = 100 × (gut weight/final body weight (g)).

### 2.7. RNA Extraction and Reverse Transcription

Total RNA was extracted from the anterior intestines (50 mg/sample) using the Micro RNA Mini Extraction Kit (Applied Biotechnology, Egypt) following the manufacturer’s protocol. The purity and concentration of RNA were measured using a nanophotometer (Implen GmbH, München, Germany). To produce 30 μL of cDNA from each sample, 1.5 μg of total RNA was used with the RevertAid cDNA Synthesis Kit (Thermo Scientific, Dreieich, Germany) following the manufacturer’s instructions.

### 2.8. Quantitative Real-Time PCR (RT-qPCR) and Data Analysis

RT-qPCR was performed using the Maxima SYBR Green qPCR kit (Thermo, Waltham, MA, USA) and the QuantStudio^TM^ real-time qPCR detection system (Applied Biosystems, Waltham, MA, USA). The expressions of *IL-1β*, *IL-10* and *IgM* genes were detected using the primer sets (Table 2). The cycling profile was as follows: an initial activation step at 95 °C for 10 min, followed by 40 cycles of 95 °C for 30 s, 60 °C for 1 min as an annealing step and 72 °C for 1 min as the extension step. The obtained melting curve analysis verifies the specificity and identity of PCR products. Tilapia *β-actin* and elongation factor alpha (*EF1α*) genes (Table 2) were utilized as housekeeping genes for cDNA normalization. Following the method proposed by Livak and Schmittgen [32], the delta-delta Ct (2^−(ΔΔCt)^) approach was employed to determine the fold change in the expression of *IL-1β*, *IL-10* and *IgM* genes. Subsequently, the results were subjected to statistical analysis.

### 2.9. Statistical Analysis

Feed efficiency, growth performance and somatic indices data were presented as mean ± SEM. An unpaired one-way ANOVA test was used to determine significance between groups at *p* ≤ 0.05. Subsequently, a post hoc test (Tukey) was employed for multiple comparisons. One and two-way analysis of variance (ANOVA) was conducted to analyze the data on the relative expression of *IL-1β*, *IL-10* and *IgM*. All analyses were performed using GraphPad Prism^®^ 10 Software (version 10.5.0). The *p*-value for each data analysis was calculated. A heat map was generated using GraphPad Prism 10 Software (version 10.5.0). On a colour scale ranging from blue, which denotes lower expressions, to yellow, which denotes higher expressions, colour values represent log^2^ (fold change). Additionally, hierarchical cluster analysis with a heat map was carried out using the expression function within Heatmapper at https://heatmapper.ca/expression/ (accessed on 18 December 2025).

## 3. Results

### 3.1. UPLC-ESI-MS/MS Analysis of S. terebinthifolius Ethanolic Leaf Extract

The ultra-performance liquid chromatography-electrospray ionization-tandem mass spectrometry (UPLC-ESI-MS/MS) analysis was conducted on the extract of *S. terebinthifolius*, utilizing both positive (ES+) and negative (ES-) ionization modes (Supplementary Figures S1 and S2). The base peak intensity (BPI) chromatograms revealed a series of peaks corresponding to various metabolites, with retention times ranging from 0.72 to 31.34 min in ES+ mode and 0.75 to 31.11 min in ES- mode. The chromatographic parameters, including retention time, absolute area, percentage of total area, peak width, and height, are detailed in Supplementary Tables S1 and S2 for the major detected peaks.

The mass spectral data associated with these peaks enabled the tentative identification of compounds based on neutral masses, adduct patterns, and comparisons with literature reports on *S. terebinthifolius* metabolites. The identified compounds are summarized in Table 3, which includes the peak number, retention time, polarity, major observed *m*/*z*, calculated neutral mass, assumed adduct, proposed compound name, and reference.

### 3.2. Performance Metrics: Growth, Feed Efficiency, and Somatic Parameters

At 30 and 60 days after starting the feeding experiment, the feed efficiency, growth performance, and somatic indices of *O. niloticus* were evaluated and summarized in Table 4. The results showed that all treated groups experienced a significant increase in final body weight, weight gain, weight gain percentage, feed intake, growth rate, and specific growth rate (*p* ≤ 0.05) compared to the control group, with the concentration and the time increasing. Consequently, these improvements were reflected in the feed conversion ratio, which was significantly better (*p* ≤ 0.05) in all supplemented groups compared to the control group as the dosage and time increased. Additionally, no mortalities or negative effects were observed in any of the groups (C, T0.5%, T1% and T2%) throughout the feeding period (60 days), and all groups had a 100% survival rate.

### 3.3. Expression Profiles of Immune-Related Genes

**Figure 2:**
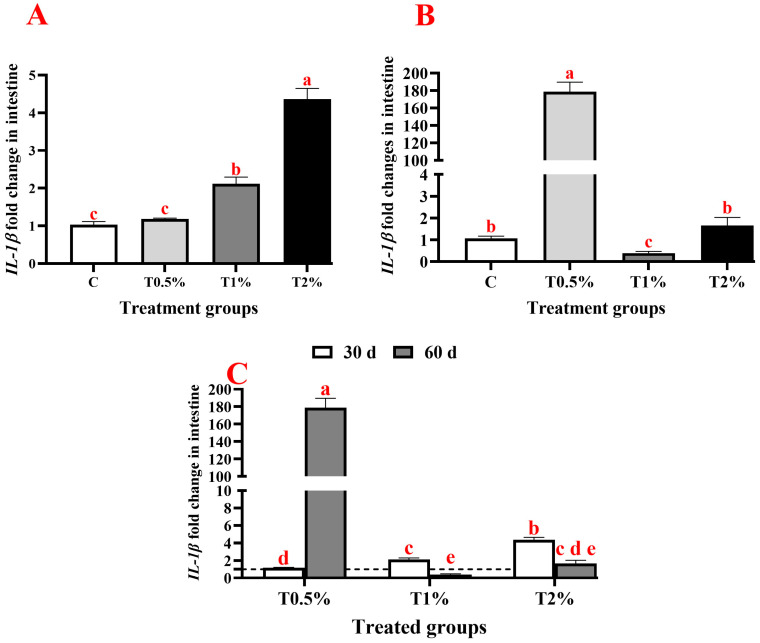
Expression profiles of *IL-1β* gene in the anterior intestine of Nile tilapia (*Oreochromis niloticus*) after receiving different concentrations of *Schinus terebinthifolius* Raddi ethanolic leaf extract (T0.5%: 0.5%, T1%: 1%, and T2%: 2%) at 30 days (**A**), 60 days (**B**), and at both 30 and 60 days post-feeding (**C**) with the dash line represent the control level. Expression levels were normalized against the expression of both *β-actin* and *EF1α* genes. The data are presented as means ± SEM. The significant differences (*p* < 0.05) are shown by different lowercase letters.

**Figure 3:**
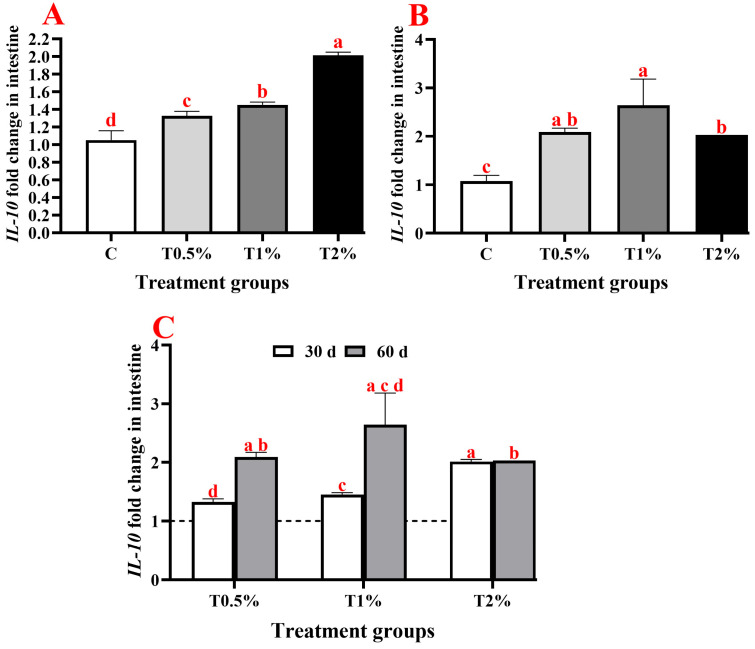
Expression profiles of the *IL-10* gene in the anterior intestine of Nile tilapia (*Oreochromis niloticus*) after receiving varying concentrations of *Schinus terebinthifolius* ethanolic leaf extract (T0.5%: 0.5%, T1%: 1%, and T2%: 2%) at 30 days (**A**), 60 days (**B**), and at both 30 and 60 days post-feeding (**C**) with the dash line represent the control level. Expression levels were normalized against the expression of both *β-actin* and *EF1α* genes. Data are expressed as means ± SEM, with n = 9. Different lowercase letters indicate the significant differences (*p* < 0.05).

**Figure 4:**
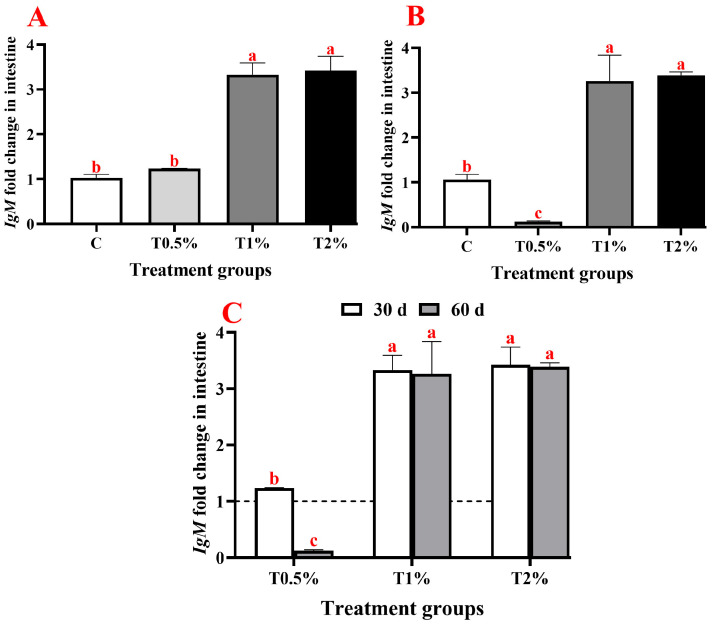
Expression profiles of *IgM* gene in the anterior intestine of Nile tilapia (*Oreochromis niloticus*) after receiving varying concentrations of *Schinus terebinthifolius* ethanolic leaf extract (T0.5%: 0.5%, T1%: 1%, and T2%: 2%) at 30 days (**A**), 60 days (**B**), and at both 30 and 60 days post-feeding (**C**) with the dash line represent the control level. Expression levels were normalized against the expression of both *β-actin* and *EF1α* genes. Data are expressed as means ± SEM. Different lowercase letters indicate the significant differences (*p* < 0.05).

On day 30, adding *S. terebinthifolius* leaf extract to the fish diet resulted in a significant upregulation of gene expressions of pro-inflammatory cytokine (*IL-1β*), anti-inflammatory cytokine (*IL-10*), and the immunoglobulin (*IgM*) in the intestine of *O. niloticus* compared to the control group. The *IL-1β* gene showed significant upregulation in all examined groups except for the first group (T0.5%), which received 0.5% of the extract, showing a statistically insignificant difference compared to the control group (Figure 2A). Additionally, there was a significant up-regulation in the expression of the *IL-10* gene in all treated groups, which was significantly upregulated with the increase in extract concentration in the incorporated feed (Figure 3A). Furthermore, almost the same trend was noticed in *IgM* gene expression, which was significantly upregulated in all treated groups (except for T0.5%) compared to the control. However, there were no significant differences between the second and third groups that received 1% and 2% of the ethanolic leaf extract (Figure 4A). On day 60 of the feeding experiment, the *IL-1β* gene expression in the T0.5% group was significantly upregulated, reaching 177-fold higher than that of the control group. Nonetheless, the expression levels were significantly downregulated in both T1% and T2% groups, with negligible variation from the control group (Figure 2B,C). The *IL-10* gene expression was significantly upregulated in all *S. terebinthifolius* leaf extract-administered groups (Figure 3B,C). Similarly, both T1% and T2% groups had significantly upregulated *IgM* gene expression, although there were no significant differences between them. However, it was downregulated in the T0.5% group compared to all other groups (Figure 4B,C).

When all assessed genes at two checkpoints were compared together, it was found that the expression of the *IL-1β* gene after 60 days of feeding in T0.5% demonstrated the highest significant increase across all treated groups. This is followed by the expression of the *IL-1β* gene in T2% at 30 days of feeding, which differed significantly from all other groups except for the expression of *IL-10* T2% at 30 days of feeding and the expression of *IgM* at both examined time points in T1% and T2%.

### 3.4. Heat Map Visualization of the Studied Genes

**Figure 5:**
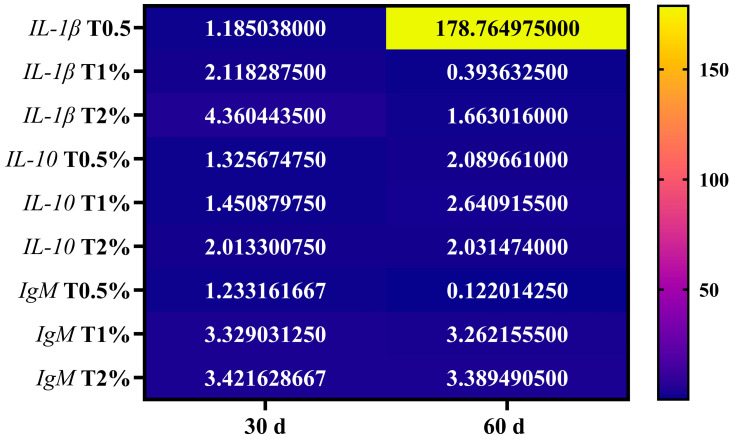
The heat map illustrates the results of quantitative real-time PCR for mRNAs of immune-related genes (*IL-1β*, *IL-10*, and *IgM*) in the anterior intestine of Nile tilapia (*Oreochromis niloticus*) after receiving different dosages of *Schinus terebinthifolius* Raddi ethanolic leaf extract (T0.5%: 0.5%, T1%: 1%, and T2%: 2%) at both 30 and 60 days post-feeding experiment commencement. The heat map was generated using GraphPad Prism 10 Software (version 10.5.0), with a colour value representing log^2^ (fold change).

As the expression of the *IL-1β* gene was greatly upregulated (178.76-fold change), it was presented as a yellow colour in the heat map. However, other genes in both 30 and 60 days were presented in blue colour as their fold change value were markedly lower than that of the *IL-1β* gene (Figure 5).

### 3.5. Hierarchical Cluster Analysis with Heat Map Visualization of the Studied Genes

**Figure 6:**
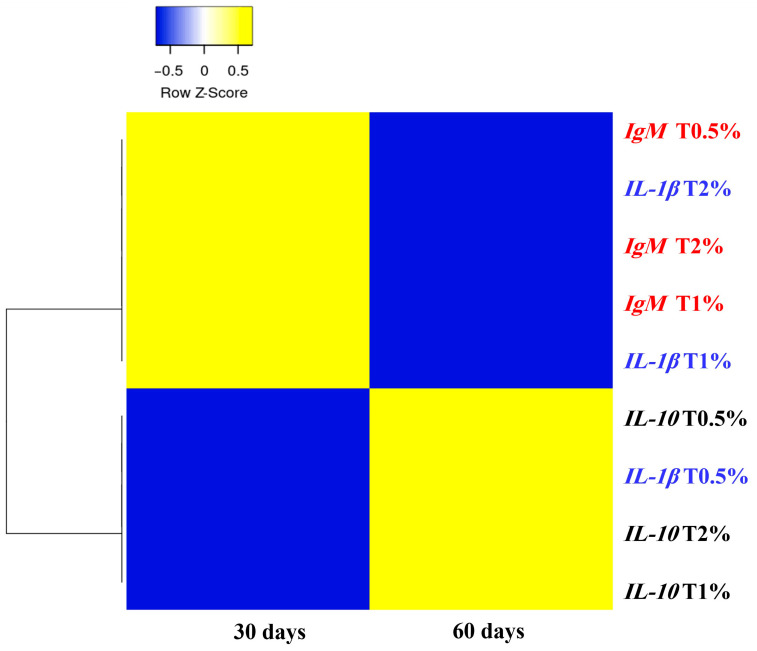
The heat map displays the hierarchical clustering of three genes—*IL-1β*, *IL-10*, and *IgM*—in the anterior intestine of Nile tilapia (*Oreochromis niloticus*) after receiving varying concentrations of *Schinus terebinthifolius* ethanolic leaf extract (T0.5%: 0.5%, T1%: 1%, and T2%: 2%). The expression values for each row are shown on a colour scale ranging from blue (low expression) to yellow (high expression). The genes are clustered using the expression function in Heatmapper, which is based on the Euclidean correlation coefficient distance matrix and average linkage hierarchical clustering.

The similarities and differences in co-expression among genes across different treatments at two time points (30 and 60 days) were illustrated using a heat map with a row-clustering dendrogram. It was observed that the values for the *IL-1β* gene expression for T1% and T2% and the *IgM* gene expression for all treatments were found to be clustered together in one main cluster, whereas the *IL-10* gene expression value for all treatments was found to be associated with the *IL-1β* gene expression value for T0.5% in another main cluster (Figure 6).

## 4. Discussion

Chemotherapies have long been used in aquaculture to fight infections and enhance the health and performance of aquatic animals. However, these chemosynthetic products can suppress natural immunity, lead to bacterial resistance, and result in environmental hazards [46]. In many countries, strict food safety and quality regulations have prompted a shift towards using natural compounds like medicinal herbs and their derivatives as alternatives to chemotherapeutics [47]. The application of phytogenic extracts in aquaculture supports fish health and prevents disease through multiple mechanisms, such as immunostimulant, growth promotion, and appetite enhancement. This broad spectrum of activity is linked to the diverse phytochemicals present, such as saponins, glycosides, phenolics, steroids, and terpenoids [1].

The incorporation of *S. terebinthifolius* extracts into fish’s diet has demonstrated potential benefits on various physiological and immunological parameters. This may likely be attributable to its rich profile of bioactive compounds (Table 5).

This study demonstrates significant improvements in growth performance, weight gain, and weight gain percentage in *O. niloticus*-fed *S. terebinthifolius* leaf extract, which are correlated with enhanced feed utilization as evidenced by a reduced FCR value. These progressive, dose-dependent improvements in final body weight, weight gain, and weight gain percentage indicate a significant positive response to the treatment over time, with the most substantial enhancements observed at the high concentration (T2%). Additionally, feed intake increased significantly with the increase in the concentration of the administered *S. terebinthifolius*, where the higher *S. terebinthifolius* doses boosted feed intake, potentially linked to improvement of gut health by its rich content of phenolic compounds, e.g., gallic acid, ferulic acid and caffeic acid. In zebra fish, gallic acid maintains the integrity of both the intestine and the liver. It acts as an anti-inflammatory, reduces intestinal inflammation, and restores both intestinal villus length and mucosal CD4 signals to the normal healthy levels. In the hindgut, it restrains the inflammatory cells, neutrophils, macrophages, and T-lymphocytes from clustering. Beneficial shifts in gut bacteria occur, including higher *Verrucomicrobia* levels and a restored *Firmicutes* to *Bacteroidetes* balance, which safeguard the intestinal lining. It also triggers production of gut metabolites that combat inflammation through immune regulation and antioxidant actions [48]. Improvement of FBW, WG, SGR and FCR was recorded in *C. carpio* fed on gallic acid feed additive. Gallic acid significantly enhanced the activity of key digestive enzymes (protease, lipase, and amylase), indicating improved nutrient digestibility and utilization. Serum levels of liver enzymes (AST, ALP, and LDH) were reduced, indicating better hepatic health and reduced tissue damage [50]. Dietary supplementation with ferulic acid at 400 mg/kg for 90 days demonstrably reduced the adverse intestinal effects induced by oxidized fish oil in *O. niloticus*. Ferulic acid improves oxidative stress markers, digestive enzyme (protease, amylase and lipase) activity in the intestine, partially restores intestinal villus morphology, and modulates the gut microbiota towards a more balanced state, which improves growth performance and feed utilization [61]. Caffeic acid improved the activity of digestive enzymes (amylase, lipase, and pepsin) in *H. huso* [57].

Feed conversion ratio (FCR) decreased significantly with the increase in the concentration and the timing of the administered *S. terebinthifolius* extract in the current investigation, reflecting enhanced feed efficiency across increasing concentrations, which nearly duplicated the specific growth rate in the higher dose (T2%) from 0.92 ± 0.0% at day 30 to reach 1.6 ± 0.0% at day 60 post-feeding, which confirms that *S. terebinthifolius* has a reliable growth-promoting effect over time. This enhancement in the fish growth performance is typically linked to efficient feed utilization, which reflects the health of intestinal digestion [65]. Additionally, supplementing *O. niloticus* feed with 4% *S. terebinthifolius* powder for 40 days improved feed efficiency; FCR decreased by 16%, and growth rate SGR increased by 16% compared to the standard diet [66]. Caffeic acid upregulated the gene expression of growth hormone and insulin-like growth factor in *H. huso* [57]. Growth hormone plays a crucial role in regulating metabolism, primarily by stimulating processes such as protein synthesis, cellular RNA formation, and amino acid transport. Insulin-like growth factors secretion and regulation are growth hormone-dependent; this is critical for activation of cellular growth pathways, initiation of cell proliferation and differentiation essential for growth [57,67,68,69,70]. *P*-coumaric acid was recorded to enhance SGR and WG and decrease FCR in *C. carpio* [71]. It functions both as a palatability enhancer and as a potential digestive aid, promoting greater feed consumption. Its interaction with gut microbiota could improve diet digestibility and nutrient absorption efficiency across the intestinal epithelium [72,73]. Quercetin improved WG, SPG and FCR in snakehead fish (*Channa argus*) supplemented with 300 mg kg^−1^ [56]. Increasing ferulic acid concentration in the diet leads to adverse effects on SGR and FC in juvenile hybrid grouper (*Epinephelus fuscoguttatus*♀ × *E. polyphekadion*♂) [74]. Dietary administration of gallic acid for 60 days significantly enhanced *O. mykiss* growth performance. It elevated FW, WG, SGR, and daily WG. The addition of 450 mg/kg led to the highest growth indices, whereas feed utilization was the most efficient in the 300 mg/kg fed group [49].

Regarding the total length in the current investigation, it exhibited a dose-dependent response with the highest dose yielding the most favourable outcomes. However, these superior results were most pronounced following the prolonged feeding duration of two months (17.1 ± 0.2 cm), as opposed to the shorter one-month feeding (14.98 ± 0.1 cm). This pattern suggests that prolonged exposure enhances linear growth, likely through improved nutrient utilization and protein accretion, consistent with the additive’s role in promoting muscle development over lipid storage.

The condition factor initially increased across treatments during the first 30 days, with the medium dose identified as optimal for enhancing relative plumpness. However, by day 60, values declined below control levels in all treated groups; T0.5%, T1% and T2% (stabilized at optimal levels (2.1 ± 0.0)). Additionally, both viscero-somatic and hepato-somatic (unpublished data) indices decreased significantly at day 60 post-feeding in T2%. Teleosts typically store fat as adipose tissue in the abdominal viscera, hepatopancreas (liver), or in their muscle [75]. However, the majority of fat is stored in either the liver or in the visceral organs, since 90 to 95 per cent of the Nile tilapia muscle is white muscle [76]. VSI is largely affected by the level of fat deposition in the body. Fish fed diets with high lipid content showed an elevation in VSI value due to increased visceral lipid buildup [77]. The temporal shift in condition factor aligns with a progressive reduction in body fat reserves and a leaner phenotype, which is advantageous in aquaculture for maximizing edible muscle yield and minimizing processing waste. Elevated serum lipid levels are broadly acknowledged as markers of declining physiological condition in aquaculture species [78]. Hyperlipidemia can accelerate the buildup of cholesterol and triglycerides in the liver, which can lead to fatty liver disease [79], causing poor growth, increased vulnerability to disease, and greater mortality [80]. In the *O. mykiss* female, quercetin aids in the prevention of fatty liver disease. It lowers lipid accumulation, reducing oxidative stress, blocking cell death pathways, and inhibiting hepatic steatosis caused by a high-fat experimental diet [55]. In *D. rerio*, ethyl gallate reduces inflammatory responses and fat accumulation in blood vessels. It reduces lipid deposition and macrophage presence in forming plaques, and ethyl gallate prevents atherosclerosis in its early stages [52].

Caffeic acid phenethyl ester regulates lipid metabolism and reduces fat deposition by elevating PPARγ expression, a central regulator of adipocyte differentiation, while suppressing triglyceride synthesis genes (dga1, dga2) and activating lipid catabolic pathways. It enhanced glucose utilization through upregulation of glut4, gk, pk expression and downregulation of pdk4 expression. In the hepatopancreas, it similarly decreased lipid accumulation by inhibiting dga1 and dga2 and stimulating lipolytic and β-oxidation genes (atgl, cpt1b), together with beneficial shifts in glucose metabolism genes. These actions promote adipocyte hyperplasia and enhance systemic glucose and lipid catabolism, thereby protecting against hepatic steatosis [58]. Oleanolic acid markedly decreased the formation of hepatic lipid droplets, total cholesterol, and triglycerides caused by a high-fat meal [64]. *S. terebinthifolius* oil enhanced the condition factor and survival rate in Mato Grosso fish *(Hyphessobrycon eques*) without affecting feed consumption [77]. In the current study, the gastro-somatic index at 30 days of feeding only exhibited a significant increase in T2%; however, by day 60, T2% elicited a marked reduction below all other treatments and the control. As this index often relates to gastrointestinal mass or feeding intensity, its decline may indicate efficient digestion or reduced gut fill, complementing the overall shift toward leaner body composition.

The administration of *S. terebinthifolius* extract resulted in modulation of *IL-1β* gene expression in intestinal tissues, which varied with dosage and treatment duration, highlighting its role in regulating pro-inflammatory pathways. At day 30 post-feeding, T1% and T2% *S. terebinthifolius* extract significantly increased over the control, with 2% being significantly elevated above T1%. By day 60 post-feeding, the T0.5% induced a substantial upregulation, exceeding that of other treated groups at both checkpoints (30 d and 60 d). However, T1% exhibited a significant downregulation below the control and other treated groups at both checkpoints (30 d and 60 d), while T2% returned to control levels, surpassing the T1% 60-day, but remaining markedly lower than T0.5%. This suggests a biphasic pattern, which may be related to continuous cumulative bioactive interactions like gallic acid, where early small stimulations at larger dosages develop into noticeable alterations over longer durations. During the first checkpoint (30 d), T0.5% did not initiate notable pro-inflammatory activity, likely because bioactive levels fell short of activation thresholds, while higher doses led to steady increases, signalling early adaptation to stress or gentle inflammatory preparation. Continuous feeding for 60 days amplified this pattern, with T0.5% causing hyperactivation, likely reflecting chronic low-level stimulation leading to feedback amplification in signalling cascades, whereas T1% induced downregulation, perhaps via overcompensation or desensitization. On the other hand, T2% promoted homeostatic level resolution, suggesting a threshold-dependent shift toward anti-inflammatory dominance.

In *O. mykiss*, fed on gallic acid for 60 days, the expressions of key pro-inflammatory cytokines, *IL-1β*, *IL-8* and *TNF-α* genes were differentially modulated, where *IL-1β* showed a dose-responsive pattern. Insignificant upregulation of *IL-1β* was recorded at lower dose (300 mg/kg), while significant downregulation was observed at higher doses (450 and 600 mg/kg). The *IL-8* gene was downregulated in all groups, while *TNF-α* and *HSP70* genes were significantly upregulated in the 300 mg/kg group but downregulated at the highest dose, 600 mg/kg [49]. The *IL-8* suggested an anti-inflammatory effect of gallic acid, while *TNF-α* and *HSP70* indicated a biphasic or dose-dependent relationship that may reflect a shift from immunostimulant to immunosuppression at elevated concentrations. Gallic acid produces intestinal metabolites that reduce inflammation and have antioxidant and immunomodulatory properties. Gallic acid (65 ppm) significantly decreased inflammatory neutrophil and macrophage clustering in the intestines. It reduced the expression of T cell and macrophage-related genes, including foxp3a, lck, and mpeg, and reduced the inflammatory aggregation of lck: EGFP-labelled T cells resulting from soybean meal. Gallic acid treatment then prevented Mpeg-EGFP positive cells from aggregating in an inflammatory manner [48].

The *IL-10* gene expression in the current investigation exhibited a consistent dose-related upregulation across both treatment checkpoints (30 d and 60 d), reflecting the extract’s capacity to strengthen anti-inflammatory response in intestinal tissues. Comparative analysis of *IL-10* and *IL-1β* expression patterns reveals an antagonistic regulatory dynamic, where *IL-10*’s progressive upregulation across doses contrasts with *IL-1β*’s biphasic fluctuations, suggestive feedback loop modulating intestinal homeostasis. While *IL-1β* displayed initial restraint at low doses followed by hyperactivation at 60 days, *IL-10* demonstrated sustained escalation, indicating the extract’s preference for enhancing anti-inflammatory pathways over pro-inflammatory ones at higher concentrations. This opposing pattern highlights the extract’s ability to adjust cytokine levels in favour of healing, minimizing harm to tissues and aiding restoration in fish during extended treatment. This may be attributed to the presence of quercetin. Intestinal expression level of *TNF-α* and *IL-8* genes in *D. rerio* downregulated by increasing quercetin additive to a certain concentration, while *TGF-β* and *IL-10* mRNA upregulated, so determining the ideal quercetin concentration is vital for efficient regulation of inflammatory cytokines expression. Quercetin likely modulates cytokine expression by inhibiting the *NF-κB* and p38 MAPK signalling pathways, a mechanism also observed in humans, where it reduces pro-inflammatory cytokine production (*TNF-α*, *IL-1β*, and *IL-6*) [81].

The *IgM* gene expression in intestinal tissues was upregulated by all doses at both checkpoints (30 d and 60 d), with the only exception being observed at 60 days, where T0.5% caused a significant decline below baseline, and its 30-day equivalent point. This selectivity suggests that low concentration and prolonged durations eventually result in immunosuppression, while optimal extract levels and/or short duration (30 days of feeding) maintain antibody-mediated defences. In another study, *C. argus* fed quercetin feed additive (50, 300 and 450 mg/kg^−1^ feed) showed a significant increase in serum IgM level after 56 days of feeding [56]. The same findings were shown in *O. mykiss* given food including 0.1% quercetin for 14 days [82].

The observed improvements in growth performance and intestinal immune modulation in *O. niloticus* in the current investigation are most likely the result of synergistic interactions among the diverse bioactive compounds identified in *S. terebinthifolius* ethanolic leaf extract. The combination of phenolic acids (gallic, ferulic, and caffeic acids), flavonoids (myricetin-O-glucoside, quercetin derivatives, and afzelin), and gallotannins (glucogallin and pentagalloylglucose) creates a multi-target effect that single compounds alone cannot achieve. Phenolic acids and flavonoids act synergistically as potent antioxidants, scavenging free radicals and boosting endogenous antioxidant enzymes [50,54,55,61,83], while gallotannins provide complementary anti-inflammatory activity by inhibiting NF-κB signalling and modulating cytokine balance [84,85]. This coordinated action explains the biphasic *IL-1β* response (controlled activation at optimal doses) and the consistent upregulation of the anti-inflammatory cytokine *IL-10*, ultimately reducing intestinal inflammation and supporting mucosal integrity. Furthermore, the synergistic enhancement of *IgM* expression by flavonoids likely improves humoral immunity and disease resistance [56,82]. These combined effects also improve nutrient absorption and feed utilization, leading to the observed dose-dependent increases in weight gain, specific growth rate, and efficiency.

## 5. Conclusions

This study presents the first comprehensive evaluation of *S. terebinthifolius* ethanolic leaf extract as a dietary supplement in *O. niloticus*. UPLC-ESI-MS/MS analysis identified a rich bioactive metabolite profile directly linked to improve in vivo growth and intestinal immune responses. The optimal inclusion level of 2% *w*/*w* and the 60-day feeding duration yielded up to 197.3 ± 3.5% weight gain, a 1.6% rise in specific growth rate, enhanced feed efficiency, leaner growth, and favourable immunomodulation that promotes humoral immunity and reduces inflammation, without adverse effects. The extract offers a promising natural feed additive for sustainable *O. niloticus* aquaculture, which is capable of simultaneously improving production performance and intestinal health. However, large-scale field feeding trials are recommended to validate these findings under commercial conditions.

## Figures and Tables

**Table 1:** Analysis of the basal diet’s ingredients and chemical constituents (dry weight, %).

Ingredients	% In Fish Ration
Fish meal (65%)	9
Soybean meal (46%)	36.85
Corn gluten (60%)	12.2
Yellow corn	19.25
Wheat bran	5.7
Fish oil	6.00
Starch	7.00
Mineral premix (without se)	2.00
Vitamin premix	2.00
Total %	100
Proximate composition % dry matter (DM)	
Crude protein (CP)	30
Crude fibre (CF)	4.8
Ash	8.2
Ether extract (EE)	6.5
* Nitrogen-free extract (NFE)	50.5

* NFE = 100 − (CP% + EE% + CF% + Ash%).

**Table 2:** Primers used for qPCR in this study.

Primer	Sequence (5′→3′)	GenBank Number/Reference
*EF1α-E1*F *EF1α-E1*R	CTACGTGACCATCATTGATGCC AACACCAGCAGCAACGATCA	[33]
*β-actin* F1 *β-actin* R1	CAGCAAGCAGGAGTACGATGAG TGTGTGGTGTGTGGTTGTTTTG	[34]
*IL-1β* F *IL-1β* R	TGCACTGTCACTGACAGCCAA ATGTTCAGGTGCACTATGCGG	[35]
*IL-10* F *IL-10* R	CTGCTAGATCAGTCCGTCGAA GCAGAACCGTGTCCAGGTAA	[36]
*IgM* F *IgM* R	AGGAGACAGGACTGGAATGCACAA GGAGGCAGTATAGGTATCATCCTC	KJ676389.1

**Table 3:** Chemical profiling and identification of bioactive constituents of the ethanolic extract of *Schinus terebinthifolius* Raddi from UPLC-ESI-MS/MS analysis.

Peak	RT (min)	Polarity	Major *m*/*z*	Neutral Mass (Da)	Adduct	Proposed Compound Name	Reference
1	0.75	ES−	169.3631	170.3704	[M−H]^−^	Gallic acid	[37]
1	0.75	ES−	331.6320	332.6393	[M−H]^−^	Glucogallin (1-O-galloyl-β-D-glucose)	[37]
2	0.92	ES−	169.3533	170.3606	[M−H]^−^	Gallic acid	[37]
3	2.07	ES−	183.4145	184.4218	[M−H]^−^	Methyl gallate	[38]
4	5.23	ES+	171.347	170.3397	[M+H]^+^	Gallic acid	[39]
4	5.24	ES−	197.4449	198.4	[M−H]^−^	Ethyl gallate	[38]
4	5.24	ES−	479.6184	480.6257	[M−H]^−^	Myricetin-O-glucoside	[37]
5	5.99	ES−	463.6466	464.6539	[M−H]^−^	Quercetin 3-O-glucoside	[37,40]
6	6.61	ES+	449.5297	448.5224	[M+H]^+^	Quercetrin (quercetin 3-O-rhamnoside)	[41]
6	6.61	ES+	303.4655	302.4582	[M+H]^+^	Quercetin	[40,42]
6	6.63	ES−	335.5286	336.5359	[M−H]^−^	Methyl digallate	[38,39]
6	6.63	ES−	599.9161	600.9234	[M−H]^−^	Galloyl quercetin-rhamnoside	[37]
7	7.05	ES−	349.5731	350.5804	[M−H]^−^	Ethyl digallate	[38]
7	7.05	ES−	431.6838	432.6911	[M−H]^−^	Afzelin (kaempferol 3-O-rhamnoside)	[43]
7	7.04	ES+	433.5647	432.5574	[M+H]^+^	Afzelin (kaempferol 3-O-rhamnoside	[43]
8	7.41	ES−	193.4379	194.4452	[M−H]^−^	Ferulic acid	[44]
8	7.42	ES−	939.7849	940.7922	[M−H]^−^	β-penta-O-galloyl-glucose	[37]
9	8.18	ES−	469.7642	470.7715	[M−H]^−^	Triterpene acid	[45]
11	8.98	ES−	455.4842	456.4915	[M−H]^−^	Oleanolic acid	[42]
12	9.22	ES−	193.5218	194.5291	[M−H]^−^	Ferulic acid	[44]
13	9.53	ES−	191.3501	192.3574	[M−H]^−^	Quinic acid	[45]
16	20.61	ES−	179.6185	180.6258	[M−H]^−^	Caffeic acid	[40,42]
17	21.06	ES−	453.8931	454.9004	[M−H]^−^	Masticadienoic acid	[45]
19	22.37	ES+	635.851	634.8437	[M+H]^+^	Trigalloylglucose	[45]
20	23.21	ES−	453.8536	454.8609	[M−H]^−^	Masticadienoic acid	[38]
21	23.46	ES+	457.8481	456.8408	[M+H]^+^	Oleanolic acid	[42]
			456.8107	455.8034			
			455.8022	454.7949			
21	23.47	ES−	453.907	454.9143	[M−H]^−^	Masticadienoic acid	[38]
22	23.9	ES−	319.7121	320.7194	[M−H]^−^	Anacardic acid (13:0)	[38]
24	24.13	ES−	319.6919	320.6992	[M−H]^−^	Anacardic acid (13:0)	[38]
26	24.62	ES−	371.8175	372.8248	[M−H]^−^	Anacardic acid (17:2)	[38]
30	26.86	ES−	373.8378	374.8451	[M−H]^−^	Anacardic acid (17:1)	[38]
32	31.11	ES−	317.3633	318.3706	[M−H]^−^	Myricetin	[45]

**Table 4:** Effects of *Schinus terebinthifolius* Raddi ethanolic leaf extract on feed efficiency, growth performance, and gastro-somatic indices of Nile tilapia (*Oreochromis niloticus*) at 30 and 60 days post-feeding experiment commencement.

Parameter	30 d				60 d			
	C	T0.5%	T1%	T2%	C	T0.5%	T1%	T2%
Survival rate	100	100	100	100	100	100	100	100
FBW(g)	54.3 ± 0.7 ^a^	54.97 ± 0.7 ^b^	56.8 ± 1.1 ^c^	66.4 ± 1.6 ^d^	84.0 ± 1.3 ^e^	88.99 ± 1.97 ^f^	99.96 ± 2.7 ^g^	106.9 ± 3.6 ^h^
WG (g/fish)	19.2 ± 0.3 ^a^	19.9 ± 0.3 ^b^	21.7 ± 0.3 ^c^	31.3 ± 0.8 ^d^	49.1 ± 0.5 ^e^	53.9 ± 1.2 ^f^	64.9 ± 1.8 ^g^	71.8 ± 2.7 ^h^
%WG	59.3 ± 1.6 ^a^	61.3 ± 1.6 ^b^	64.6 ± 1.2 ^c^	89.6 ± 0.9 ^d^	145.8 ± 2.2 ^e^	155.5 ± 2.2 ^f^	183.1 ± 2.3 ^g^	197.3 ± 3.5 ^h^
FI (g/fish)	33.6 ± 0.4 ^a^	34.0 ± 0.5 ^b^	35.3 ± 0.7 ^c^	37.8 ± 1.15 ^d^	55 ± 1.8 ^e^	59.6 ± 2.6 ^f^	62.6 ± 2.9 ^g^	70 ± 2.6 ^h^
FCR	1.84 ± 0.05 ^a^	1.79 ± 0.04 ^b^	1.66 ± 0.02 ^b^	1.24 ± 0.01 ^c^	1.1 ± 0.0 ^d^	1.2 ± 0.1 ^cd^	1.1 ± 0.0 ^d^	1.2 ± 0.1 ^cd^
GR (g/d)	0.64 ± 0.01 ^a^	0.66 ± 0.0 ^b^	0.72 ± 0.0 ^c^	1.04 ± 0.02 ^d^	1.6 ± 0.0 ^e^	1.8 ± 0.0 ^f^	2.2 ± 0.1 ^g^	2.4 ± 0.1 ^h^
SGR (%/d)	0.66 ± 0.02 ^a^	0.68 ± 0.02 ^b^	0.72 ± 0.01 ^c^	0.92 ± 0.0 ^d^	1.29 ± 0.0 ^e^	1.35 ± 0.0 ^f^	1.5 ± 0.0 ^g^	1.6 ± 0.0 ^h^
TL (cm)	14.4 ± 0.1 ^a^	14.3 ± 0.1 ^a^	13.9 ± 0.1 ^b^	14.98 ± 0.1 ^c^	15.4 ± 0.1 ^d^	16.1 ± 0.1 ^e^	16.7 ± 0.1 ^f^	17.1 ± 0.2 ^g^
CF	1.81 ± 0.01 ^a^	1.86 ± 0.0 ^b^	2.09 ± 0.02 ^c^	1.94 ± 0.01 ^d^	2.3 ± 0.0 ^e^	2.1 ± 0.0 ^c^	2.1 ± 0.0 ^c^	2.1 ± 0.0 ^c^
VSI (%)	13.7 ± 1.2 ^a^	13.3 ± 0.7 ^ab^	14.9 ± 0.8 ^ac^	14.9 ± 0.9 ^abc^	13.9 ± 0.7 ^ab^	14.4 ± 0.7 ^acb^	11.9 ± 0.7 ^ad^	10.6 ± 0.4 ^d^
GSI (%)	6.6 ± 0.3 ^a^	6.9 ± 0.4 ^a^	6.5 ± 0.5 ^ab^	8.4 ± 0.6 ^c^	6.1 ± 0.3 ^ab^	5.7 ± 0.3 ^b^	5.8 ± 0.2 ^b^	5.1 ± 0.1 ^d^

Data represented as means ± SEM. Within rows, values with different superscripts indicate that their corresponding meanings are significantly different at *p* ≤ 0.05 according to one-way ANOVA followed by Tukey multiple comparisons test. Abbreviations: FBW: final body weight, WG: weight gain, %WG: weight gain percentage, FI: feed intake, FCR: feed conversion ratio, GR: growth rate, SGR: specific growth rate, TL: total length, CF: condition factor, VSI: viscero-somatic index, GSI: gastro-somatic index.

**Table 5:** Identified compounds and their effects in previous fish studies.

Compound Name	Effect	Animal Studied	Reference
Gallic Acid	Reduces intestinal inflammation Stimulates liver PPAR signalling Beneficial shifts in gut bacteria Antioxidant	Zebrafish (*Danio rerio*)	[48]
	Improves WG, SGR, and FCR Increases the expression of: Antioxidant-related genes (SOD, CAT, GPX) Stress-related (HSP70) Immune-related (TNF-α, IL-1β) The effect is dose-dependent The optimal dose is 300 mg/kg.	Rainbow trout (*Oncorhynchus mykiss*)	[49]
	Improves FW, WG, SGR and FCR Improves digestive enzymes Improves liver health Enhances immunity: increases serum lysozyme (LYZ) activity, alternative complement pathway (ACH_50_), total immunoglobulin (Ig), myeloperoxidase (MPO) and respiratory burst (RB). Increases the activity of antioxidant enzymes (SOD, CAT, GPx) and reduces the oxidative stress marker malondialdehyde (MDA) in serum Lower levels of serum cortisol and glucose after exposure to crowding stress The effect is dose-dependent The optimal dose is 450 mg/kg.	Common carp (*Cyprinus carpio*)	[50]
Methyl gallate	Anti-carcinogenic effect	*D. rerio*	[51]
Ethyl gallate	Anti-atherosclerosis effect	*D. rerio*	[52]
Myricetin	Anti-viral effect against *Micropterus salmoides Rhabdovirus* (MSRV)	Largemouth bass (*Mcropterus salmoides*)	[53]
Quercetin	Enhanced the overall antioxidant defence system Mitigated oxidative stress	Spotted sea bass (*Lateolabrax maculatus*)	[54]
	Hepatoprotective effect against fatty liver	*O. mykiss*	[55]
	Enhances feed consumption and growth efficiency Boosts antioxidant capacity via increased enzyme activity Modulates immune function by upregulating gene expression and enzyme activity	Snakehead fish (*Channa argus*)	[56]
Caffeic acid	Enhances final weight, WG, and SGR Increases activity of digestive enzymes (amylase, lipase, pepsin) Boosts lysozyme activity, total immunoglobulin, and total protein Upregulates the expression of growth genes (GH, IGF), lipid metabolism (lipoprotein lipase), and immunity (nuclear factor interleukin-3)	Beluga (*Huso huso*)	[57]
	Adipose tissue remodelling: enhances adipocyte hyperplasia Enhances lipid metabolism: it increases lipolysis and β-oxidation in adipose and hepatic tissues Improves glucose homeostasis Reduces inflammation and hepatic steatosis	Grass carp (*Ctenopharyngodon idellus*)	[58]
	Antibacterial effect: against *Yersinia ruckeri E42*, *Listonella anguillarum SY-L24*, *S. iniae ATCC 2917*, *Edwardsiella tarda SY-ED14* and *Citrobacter* sp. *SY-C10*		[59]
	Immunostimulant of innate defence Enhances gene expression of immune and antioxidant pathways Enhances survival and protection against *A. veronii*	*O. niloticus*	[60]
Ferulic acid	Increases WG and decreases FCR Modulates antioxidant response: decreases malondialdehyde level in the serum and intestine Partially restores villus structure Enhances digestive enzyme activities Modulates microbial community	*O. niloticus*	[61]
Oleanolic acid	Anti-inflammatory and lipid-decreasing properties	*D. rerio* embryos	[62]
	Increases BW, WG, SGR and decreases FCR Decreases concentrations of atherogenic lipids (total cholesterol, triglycerides, and LDL-C) and increases cardioprotective HDL-C Hepatopancreatic protective effect (enhances antioxidant capacity and reduces oxidative damage)	Red swamp crayfish (*Procambarus clarkii*)	[63]
	Reduces hepatosomatic index in high-fat diet-fed tilapia Improves liver health Inhibits the abnormal accumulation of lipids in liver tissue	GIFT *O. niloticus* fingerling	[64]

## Data Availability

Data and materials are available upon reasonable request from the corresponding author.

## Supplementary Materials

The following supporting information can be downloaded at https://www.mdpi.com/article/10.3390/biology15060476/s1, Figure S1: Ethanolic extract of *Schinus terebinthifolius* Raddi chromatogram in positive-ionization mode obtained by UPLC-ESI-MS/MS. Figure S2: Ethanolic extract of *Schinus terebinthifolius* Raddi chromatogram in negative-ionization mode obtained by UPLC-ESI-MS/MS.; Table S1: Chromatographic Peaks in ES+ Mode; Table S2: Chromatographic Peaks in ES− Mode.

## Abbreviations

The following abbreviations are used in this manuscript:

ACH50	Alternative complement pathway activity (50% hemolytic complement)
ALP	Alkaline phosphatase
ANOVA	Analysis of variance
AST	Aspartate aminotransferase
AT	Adipose tissue
BEH	Bridged ethyl hybrid
BPI	Base peak intensity
BW	Body weight
C	Control group
C18	Octadecyl (C18) reversed-phase column
cDNA	Complementary DNA
CAT	Catalase
CF	Condition factor
Ct	Cycle threshold
Da	Dalton
DDGS	Distillers dried grains with solubles
DE	Digestible energy
dgaT0.5%/dgaT1%	Diacylglycerol acyltransferase 1/2 genes
EF1α	Elongation factor 1 alpha
EGFP	Enhanced green fluorescent protein
ES+/ES−	Electrospray ionization positive/negative mode
ESI	Electrospray ionization
ESI-MS	Electrospray ionization mass spectrometry
FBW	Final body weight
FCR	Feed conversion ratio
FI	Feed intake
FTN	Flow-through needle autosampler
FW	Final weight
g/Kg	Gram/kilogram
GH	Growth hormone
GPx/GPX	Glutathione peroxidase
GR	Growth rate
GSI/Ga-SI	Gastro-somatic index
GW	Gut weight
HDL-C	High-density lipoprotein cholesterol
HSP70	Heat shock protein 70
IBW	Initial body weight
IGF	Insulin-like growth factor
Ig/IgM	Immunoglobulin/Immunoglobulin M
IL-1β	Interleukin-1 beta
IL-6	Interleukin-6
IL-8	Interleukin-8
IL-10	Interleukin-10
kV	Kilovolt
L/h	Litres per hour
LDH	Lactate dehydrogenase
LDL-C	Low-density lipoprotein cholesterol
Lck	Lymphocyte-specific protein tyrosine kinase gene marker
ln (Ln)	Natural logarithm
LYZ	Lysozyme
*m*/*z*	Mass-to-charge ratio
MDA	Malondialdehyde
MAPK	Mitogen-activated protein kinase
MPO	Myeloperoxidase
MS	Mass spectrometry
MSRV	*Micropterus salmoides* rhabdovirus
NF-κB	Nuclear factor kappa B
*O. niloticus*	*Oreochromis niloticus*
PCR	Polymerase chain reaction
pdk4	Pyruvate dehydrogenase kinase 4 gene
Pk	Pyruvate kinase gene
PPAR/PPARγ	Peroxisome proliferator-activated receptor/gamma
QSM	Quaternary solvent manager
RB	Respiratory burst
ROI	Region of interest
RNA	Ribonucleic acid
RT	Retention time
RT-qPCR	Reverse transcription quantitative real-time PCR
SEM	Standard error of the mean
SGR	Specific growth rate
SOD	Superoxide dismutase
TGF-β	Transforming growth factor beta
TL	Total length
TNF-α	Tumour necrosis factor alpha
UPLC	Ultra-performance liquid chromatography
VSI	Viscero-somatic index
WG	Weight gain
