# Supplementary material for: Effects of Brazilian Pepper Tree (Schinus terebinthifolius Raddi) Ethanolic Leaf Extract on Growth Performance and Expression of Intestinal Immune-Related Genes in Nile Tilapia (Oreochromis niloticus)
Source: Biology (Basel). 2026 Mar 15;15(6):476. doi: 10.3390/biology15060476 (PMC13024724; doi:10.3390/biology15060476)
Supplement: Supplementary file 1 [file biology-15-00476-s001.zip › biology-4146858-supplementary.pdf]

Effects of Brazilian pepper tree (*Schinus terebinthifolius* Raddi) ethanolic leaf extract on growth performance and expression of intestinal immune-related genes in Nile tilapia (*Oreochromis niloticus*)

Eman Mohamed [1], Mahmoud Mostafa Mahmoud [1,*], Yosra M. I. El Sherry [2], Amr Abdullah [3], Soad A. L. Bayoumi [4,5], Rofida Wahman [4], Abeer M. Mahmoud [6,7,*], Mahmoud Mahrous Saed Farrag [8] and Ebtsam Sayed Hassan Abdallah [1]

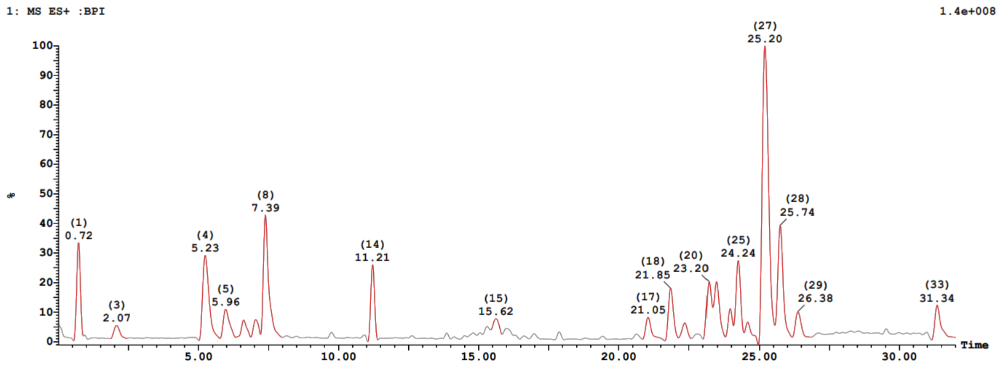

Supplementary Figure S1. Ethanolic extract of *Schinus terebinthifolius* Raddi chromatogram in positive-ionization mode obtained by UPLC-ESI-MS.

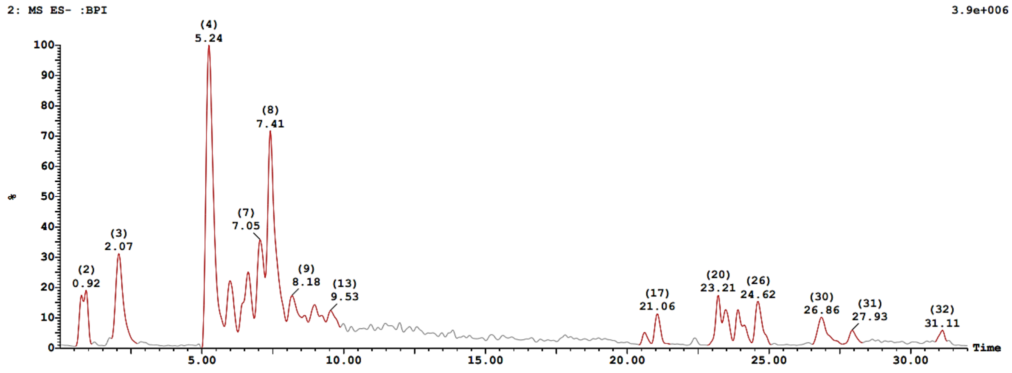

Supplementary Figure S2. Ethanolic extract of *Schinus terebinthifolius* Raddi chromatogram in negative-ionization mode obtained by UPLC-ESI-MS.

Supplementary Table S1. Chromatographic peaks in ES+ mode.

Peak	RT* (min)	Area Abs**	Area %	Width	Height
**1**	0.72	7e+006	4.9	0	4e+007
**3**	2.07	1e+006	1.06	1	6e+006
**4**	5.23	1e+007	7.75	1	4e+007
**5**	5.96	4e+006	2.79	1	1e+007
**6**	6.61	2e+006	1.54	1	9e+006
**7**	7.04	2e+006	1.36	0	9e+006
**8**	7.39	1e+007	9.25	1	6e+007
**14**	11.21	6e+006	4.07	0	4e+007
**15**	15.62	1e+006	1.01	0	6e+006
**17**	21.05	3e+006	1.91	1	1e+007
**18**	21.85	5e+006	3.71	0	2e+007
**19**	22.37	2e+006	1.24	0	8e+006
**20**	23.2	5e+006	3.83	0	3e+007
**21**	23.46	6e+006	4.69	0	3e+007
**23**	23.97	3e+006	2.01	0	2e+007
**25**	24.24	8e+006	5.74	0	4e+007
**26**	24.57	3e+006	1.97	0	1e+007
**27**	25.20	4e+007	26.73	1	1e+008
**28**	25.74	1e+007	9.31	1	5e+007
**29**	26.38	3e+006	2.29	1	1e+007
**33**	31.34	4e+006	2.86	1	2e+007

*RT: retention time; **Abs: absolute.

Supplementary Table S2. Chromatographic peaks in ES- mode.

Peak	RT* (min)	Area Abs**	Area %	Width	Height
**1**	0.75	1e+005	1.97	0	6e+005
**2**	0.92	1e+005	2.45	0	7e+005
**3**	2.07	3e+005	6.82	1	1e+006
**4**	5.24	1e+006	24.96	1	4e+006
**5**	5.99	2e+005	5.07	0	8e+005
**6**	6.63	3e+005	6.42	1	9e+005
**7**	7.05	3e+005	7.16	0	1e+006
**8**	7.41	9e+005	18.28	1	3e+006
**9**	8.18	2e+005	3.77	1	5e+005
**10**	8.63	4e+004	0.89	0	2e+005
**11**	8.98	1e+005	2.04	0	3e+005
**12**	9.22	4e+004	0.73	0	2e+005
**13**	9.53	7e+004	1.43	1	2e+005
**16**	20.61	3e+004	0.68	0	2e+005
**17**	21.06	9e+004	1.81	1	4e+005
**20**	23.21	1e+005	2.71	1	6e+005
**21**	23.47	1e+005	2.33	0	4e+005
**22**	23.9	9e+004	1.95	0	4e+005
**24**	24.13	5e+004	1.05	0	3e+005

**26**	24.62	2e+005	3.24	1	6e+005
**30**	26.86	1e+005	2.71	1	3e+005
**31**	27.93	4e+004	0.92	1	2e+005
**32**	31.11	3e+004	0.6	0	1e+005

*RT: retention time; **Abs: absolute
